# Flocculation of Clay-Based Tailings: Differences of Kaolin and Sodium Montmorillonite in Salt Medium

**DOI:** 10.3390/ma15031156

**Published:** 2022-02-02

**Authors:** Steven Nieto, Norman Toro, Pedro Robles, Edelmira Gálvez, Sandra Gallegos, Ricardo I. Jeldres

**Affiliations:** 1 Departamento de Ingeniería Química y Procesos de Minerales, Facultad de Ingeniería, Universidad de Antofagasta, P.O. Box 170, Antofagasta 1240000, Chile; yeison.nieto.mejia@ua.cl; 2 Faculty of Engineering and Architecture, Arturo Prat University, Iquique 1100000, Chile; notoro@unap.cl (N.T.); chichined@gmail.com (S.G.); 3 Escuela de Ingeniería Química, Pontificia Universidad Católica de Valparaíso, Valparaíso 2340000, Chile; pedro.robles@pucv.cl; 4 Department of Metallurgical and Mining Engineering, North Catholic University, Antofagasta 1270709, Chile; egalvez@ucn.cl

**Keywords:** seawater flocculation, clay-based tailings, kaolin and Na-montmorillonite, fractal aggregates, mineral processing

## Abstract

Complex gangues and low-quality waters are a concern for the mining industries, particularly in water shortage areas, where the closure of hydric circuits and reduction in water use are essential to maintain the economic and environmental sustainability of mineral processing. This study analyzes the phenomena involved in the water recovery stage, such as sedimentation of clay-based tailings flocculated with anionic polyelectrolyte in industrial water and seawater. Flocculation–sedimentation batch tests were performed to ascertain the aggregate size distribution, the hindered settling rate, and the structure of flocs expressed through their fractal dimension and density. The aggregates’ properties were characterized by the Focused Beam Reflectance Measurement (FBRM) and Particle Vision Microscope (PVM) techniques. The impact of the type of water depends on the type of clay that constitutes the suspension. For quartz/kaolin, the highest performance was obtained in industrial water, with bigger aggregates and faster settling rates. However, the tailings composed of quartz/Na-montmorillonite reversed this trend. The type of water impacted the efficiency of primary-particle aggregation. The trials in industrial water generated a portion of non-flocculated particles, which was observed through a bimodal distribution in the unweighted chord-length distribution. This behavior was not observed in seawater, where a perceptible fraction of non-flocculated particles was not found. The additional cationic bonds that offer seawater favor finer primary-particle agglomeration for all tailings types.

## 1. Introduction

Mining tailings are residual suspensions that come from froth flotation stages and are made up of non-valuable minerals, such as silicates and phyllosilicates. These pulps have water contents close to 70 wt%; therefore, they must be thickened to recover part of the water resource and produce a thickened pulp that can be safely stored. For this, the pulp is fed to the feed-well of thickeners, where it is mixed with chemical reagents, especially soluble polymers of high molecular that can adhere to several particles simultaneously, forming aggregates that settle by gravity [1]. The settling rate is a parameter of most interest to process engineers, since it is directly related to the amount of water recovered at this stage [2]. The phenomena that determine the efficiency of particle flocculation depends on the physicochemical properties of the flocculant (molecular weight, structure, functionality) and its operational handling (points of dosage, dilution, dosage), water chemistry (pH, salinity), thickening technology (e.g., thickener type, feed well), and mineralogical characteristics (e.g., fines ore, clays) [3,4].

**Figure 1:**
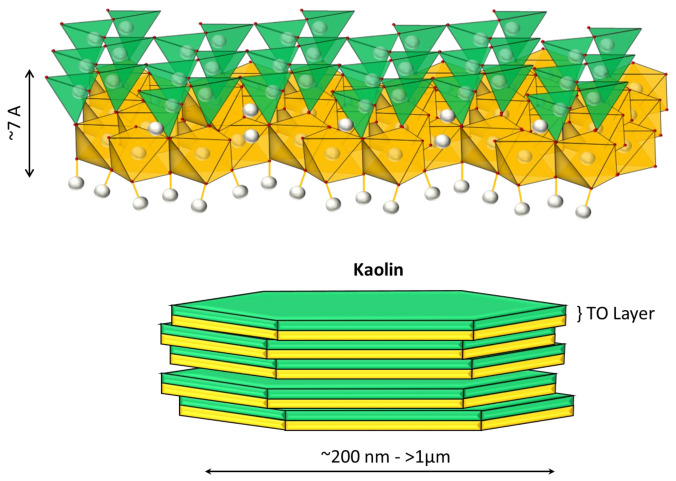
Kaolinite structure. The green sheet indicates the tetrahedral silica layer (T), and the yellow sheet represents the octahedral alumina layer (O).

**Figure 2:**
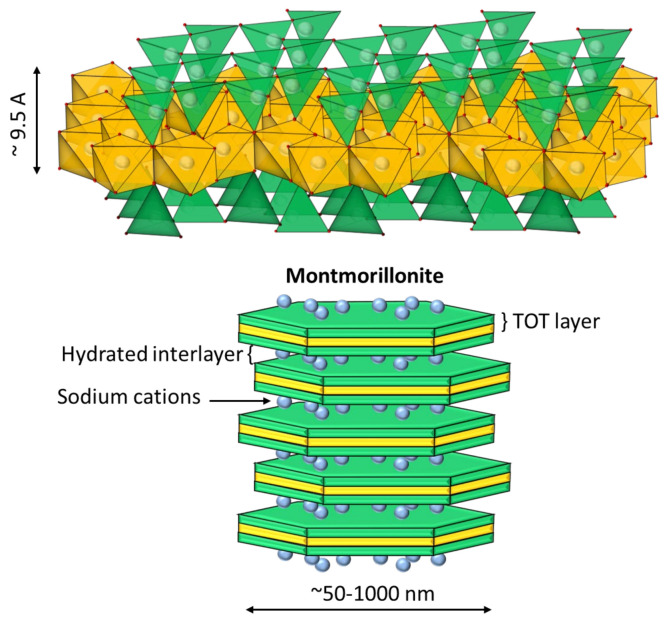
Structure of Na-montmorillonite. The green sheet indicates a tetrahedral silica layer (T), the yellow sheet indicates an octahedral alumina layer (O), and the blue spheres represent sodium cations.

A particular concern for operators is dealing with clays that are frequently associated with valuable minerals (for example, copper ores). These particles have a complex structure and affect practically all the unit stages of mineral processing, including flocculation and sedimentation phenomena [5]. Two phyllosilicates of interest in mining are kaolinite and Na-montmorillonite. Kaolinite of the composition Al_2_Si_2_O_5_(OH)_4_ is a non-swellable clay in freshwater, whose structure is composed of an octahedral sheet of aluminum hydroxide and a tetrahedral sheet of silica joined to form a basic unit of 1:1 repeat (see Figure 1). This clay has two surfaces that are crystallographically different: the faces, which have an anionic charge, and the edges, which vary their charge (anionic or cationic) depending on the pH, product of the protonation or deprotonation of the aluminol groups (Al-OH), and silanol (Si-OH) in the exposed planes with hydroxyl termination [6]. On the other hand, Na-montmorillonite is swellable in freshwater. Its complex structure consists of an octahedral sheet of alumina and two tetrahedral sheets of silica, which are joined to form a basic unit of repeating layers in a 2:1 ratio (see Figure 2) [7]. The central layer contains octahedrally coordinated Al and Mg in oxides and hydroxides, and is surrounded by two outer layers formed by tetrahedrally coordinated silicon oxides. In this structure, the water molecules enter the middle of the layers and cause swelling and modification in the mineral structure [8]. This clay has high chemical stability, a large surface area, and a high ion-exchange capacity, due to its weakly bound octahedral sheets. The increased swelling in fresh water makes sedimentation slow due to increased volumetric concentration. The swelling effect of sodium montmorillonite is reduced in a saline medium [9]. Part of the cations present in the solution migrate towards the interior of the particles, being located inside the inter-laminar layers, which have an anionic charge. The subsequent reduction in electrostatic repulsion reduces the separation distance between sheets, which prevents the entry of water molecules into the clay [10].

The particles’ electrical charge can be represented by the zeta potential, which indicates the degree of electrostatic repulsion between two surfaces. A high zeta potential value confers stability to the system, with particles that resist agglomeration. However, attractive forces can overcome repulsion when the magnitude of zeta potential is lowered and the pulp becomes unstable with flocculated particles. Empirical evidence has shown that the zeta potential of kaolin and montmorillonite is strongly influenced by the pH and salinity of the medium. In general, raising the salinity leads to a reduction in the zeta potential. This is due to the electrostatic shielding of the ions that compresses the ionic cloud surrounding the particles’ surface. On the other hand, the pH modifies the functional groups that are arranged on the particle surface, causing states in which there are no electrical charges (point of zero charge), as happens with kaolinite at a pH close to 3 [6]. However, at copper sulfide pHs relevant for the mining industry (pH > 7), zeta potential measurements reflect that the surface’s charge is anionic; therefore, the stability of the particles prevents their agglomeration with slow sedimentation. In this sense, it is strictly necessary to add chemical reagents that favor the formation of large agglomerates with fast-settling structures.

The presence of cations can promote flocculation by forming extra bonds between the anionic polyelectrolyte and the anionic faces of quartz and/or clay minerals. This is at pH conditions typical in the mineral processing of copper ore, such as chalcopyrite (pH above 7). The cationic bridges improve the adsorption density of the reagent. However, high salinity reduces the radius of gyration of polyelectrolytes, since cations shield the electrical charge of anionic functional groups that keep molecules in solution extended, limiting their ability to form polymeric bridges [11]. The competition of these phenomena can generate different results in flocculation, depending on the environment’s conditions. This could be beneficial or harmful. For example, Jeldres et al. [12] studied the impact of sodium chloride on the yielding and viscoelastic behavior of flocculated kaolinite slurries at natural pH. The authors obtained optimal flocculation at 0.001 M NaCl, which generated a higher settling rate and yield stress. Castillo et al. [13] used chemometric methods to analyze the impact of solids content, flocculant dose, shear rate, and kaolinite content on sedimentation of mining tailings and clarified water turbidity. Their results showed that the most critical variables to define turbidity were shear rate and flocculant dosage. At the same time, clay tailings were mainly influenced by solids concentration and shear rate during mixing. The lowest values of sedimentation rate were presented in saline media, which was explained by the coiling of the flocculant in the solution. Gumaste et al. [14] investigated two types of electrolytes, NaCl and SrCl_2_, in the sedimentation of sodium bentonite (90% Na-montmorillonite). The authors obtained an increase in suspension dehydration by a factor of 87 when the ionic strength of the sedimentation fluid increased from 0.1 to 250 mM. Similarly, Shaikh et al. [15] showed that electrolytes and their valence improve the sedimentation of swelling clays. Liu et al. [16] studied the kaolinite, bentonite, and illite sedimentation in saline media, using a negatively charged copolymer of acrylamide and sodium acrylate. The results showed increased salinity reduced kaolinite sedimentation, but the salinity slightly accelerated the settling rate for montmorillonite and illite. Ji et al. [17] investigated the sedimentation of clay-rich coal tailings in saline solutions in the presence of three flocculants: polyacrylamide (PAM), Magnafloc 1011 (acrylamide-acrylate copolymer), and Al (OH) 3-PAM. The Magnafloc 1011 exhibited the best sedimentation performance in saline water. The reduction in electrostatic repulsion between the negatively charged particles and anionic flocculants led to more effective polymer-bridging interactions.

To date, few studies address the flocculation of clay-based tailings with relevant systems for the mining industry. Some works that introduce microscopic characterizations stand out, directly investigating properties of the aggregates, such as size and fractal dimension. Leiva et al. [18] analyzed the temporal evolution of the aggregate structure of mining tailings with the presence of kaolin. The authors flocculated the mineral with a high molecular weight anionic polyacrylamide in seawater. Under more intense mixing and a prolonged flocculation period, the apparent decline in Df was concluded to reflect an increased contribution from the presence of aggregate fragments, i.e., broken-off branches of aggregates with a high-aspect ratio and a low effective porosity. In situ imaging of the aggregates at different reaction times supported this. Ramos et al. [19] analyzed the effect of pH on the flocculation of synthetic tailings, finding that at highly alkaline conditions (pH> 10.3), the efficiency of the process drops drastically due to the precipitation of magnesium ions. Later, Jeldres et al. [20] proposed to remove magnesium cations from seawater using a mixture of alkalizing agents, which significantly improved settling rates.

Currently, no systematic reports analyze the behavior of swelling clays, especially comparing the type of water, when these present low and high ionic charges. This research aims to analyze the structural properties of clay tailings aggregates, comparing systems with kaolin and sodium montmorillonite contents. The size distribution of the aggregates was obtained using the Focused Beam Reflectance Measurement (FBRM) technique, which obtained structural properties of aggregates, such as fractal dimension and density. This was developed by relating the size of the aggregates to the hindered settling rate [21]. The numerical outcomes for floc structure were supported by imaging obtained with the Particle Vision and Measurement (PVM) probe. The study was conducted at pH 8, considering that this is a typical pH used in the copper mining industries that use seawater in their operations. The present results are helpful for the current knowledge on clay tailings flocculation, especially with complex, swelling clays. The scope of the study involves industries that use both freshwater and seawater in their operations.

## 2. Materials and Methods

### 2.1. Materials

**Figure 3:**
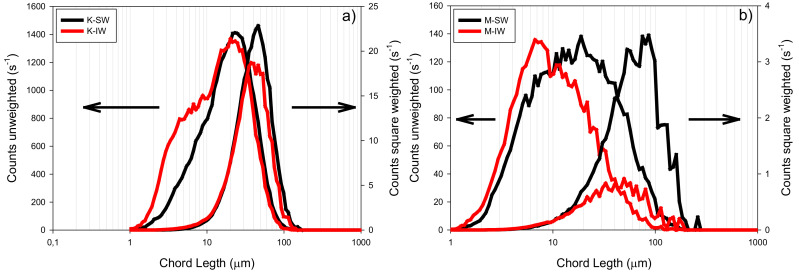
Unweighted and square-weighted chord-length distribution from (**a**) kaolin (K), and (**b**) sodium montmorillonite (M) in seawater (SW) and industrial water (IW).

The quartz particles were purchased from Donde Capo (Santiago, Chile). Firstly, 100% of the particles used were sieved under a # 270 mesh (ASTM standard). The quartz density was 2600 kg/m^3^. According to X-ray diffraction (XRD) with TOPAS software, the quartz content (Q) was higher than 99% by weight. Kaolin (K) and montmorillonite (M) particles were purchased from Ward’s Science Clay Spur, WY, USA. Figure 3 shows the chord-length distributions in unweighted (offering greater sensitivity in the range of fines) and square-weighted (influenced by the contribution of the aggregates of coarse particles) modes for kaolin (Figure 3a) and montmorillonite (Figure 3b). These data for seawater (SW) and industrial water (IW) were obtained through the FBRM technique. The change in water quality (from IW to SW) meant a shift to the right in all chord-length distributions (CLD) and growth in the maximum height weighted quadratically. This indicates that SW presents fewer fine particles (<20 µm) and more numerous aggregates between (20–90 µm) and (20–140 µm) for K and M suspensions, respectively. An increase in the salt concentration favors the coagulation of the particles, since the presence of cations reduces the ionic cloud surrounding the particles’ surface [22].

**Figure 4:**
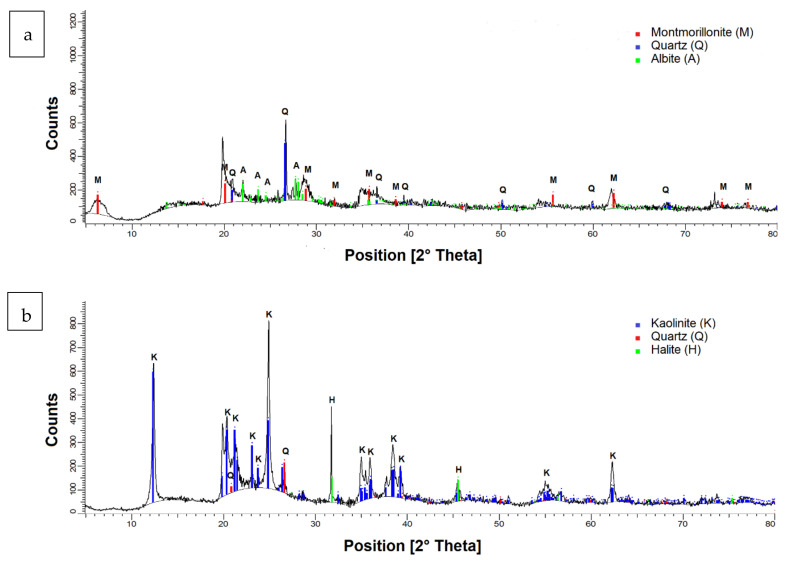
Diffractograms (**a**) sodium montmorillonite and (**b**) kaolin.

Both clays had a density of 2600 kg/m^3^. The mineralogical composition was determined by X-ray diffraction (XRD) using a Bruker brand X-ray diffractometer model D8 ADVANCE. The wavelength λ (Cukα) was 1.5406 Å. The 2θ angle studied was from 5° to 80°. The presence of mineralogical components was presented using the Powder Diffraction File of ICDD (International Center for Diffraction Data). The diffractogram shown in Figure 4a confirms the existence of montmorillonite, quartz, and albite minerals, while the diffractogram presented in Figure 4b confirms the presence of kaolinite, quartz, and albite minerals.

An anionic polyacrylamide of high molecular weight, provided by SNF Chile S.A., was used as a flocculant (SNF 704). This reagent has a molecular weight of 18 × 10^6^. A stock solution of the solid flocculant was prepared by continuous stirring for 24 h at a concentration of 1 g/L. Subsequently, a part of the stock solution was diluted to a 0.1 g/L for settling tests and flocculation kinetics. The flocculant doses were expressed as grams of flocculant per tonne of dry solids (g/t).

Seawater (SW) and industrial water (IW) were synthetically prepared. Table 1 shows the composition of SW. The IW was prepared by adding 0.005 M of CaCl_2_, 0.01 M of NaCl, and 25 g/t of Al_2_(SO_4_)_3_ as a coagulant. Both water qualities’ basic physical and chemical properties were determined using a HANNA H19829 multiparameter and an Anton Paar DMA 35 portable density (see Table 2). Analytical grade sodium hydroxide (NaOH) (greater than 98%) was used as a pH regulator. All chemical reagents were of ultra-high purity (Merck, Darmstadt, Germany).

### 2.2. Flocculation of Clay-Based Tailings

Synthetic tailings were prepared using 300 g of the kaolin-quartz (KQ) and montmorillonite-quartz (MQ) mixture in SW and IW, with a total solids concentration of 8 wt% (mixture of 90 wt% quartz and 10 wt% clay). These colloidal suspensions were prepared in a flocculating vessel, with a conical end of 1 L capacity and 100 mm in diameter, with a valve in the lower part to pour the suspensions into test tubes. The suspensions were vigorously mixed for 15 min with the aid of an up-flow polytetrafluoroethylene (PTFE) blade located 20 mm above the bottom of the container, and with a control IKA^®^ EuroStar 60 digital stirrer. Then the agitation rate was decreased to 180 rpm, and the flocculant dose of SNF 704 was added to the flocculating cup. The suspensions were prepared to develop batch sedimentation tests and flocculation kinetics.

The batch sedimentation tests were carried out after mixing the suspension with the flocculant for 20 s. This was conducted in a device constituted by a closed cylindrical column of 1000 mL volume with an internal diameter of 35 mm. The suspensions tested involved various doses of flocculant (8–34 g/t). After homogenization, the suspensions were immediately set to settle. After this, the suspension concentration was homogenized, and the initial settling rate was measured using a video camera and subsequent analysis of videos.

The particle Track G400 obtained the flocculation kinetics with FBRM technology from Mettler Toledo. The FBRM is a standard measurement technique used to measure and monitor aggregate formation and evolution in real time. This method makes it possible to analyze the mechanisms of growth, flocculation, breakage, and change in the shape of flocs through chord-length distribution conditioned by the particles’ concentration, shape, and size. The FBRM consists of a processing unit and a probe with a rotating lens that provides a laser beam focused on the external surface of a sapphire window (14 mm in diameter), responsible for scanning a circular path at a fixed speed (2 m/s for the experiments performed). When the laser beam encounters suspended solids, it produces a pulse of reflected light back to the probe window, inducing a signal peak until the particle leaves the beam’s path. The product of the tangential velocity of the spinning laser (2 m/s) and peak duration is defined as chord length, which can also be defined as an intercepted segment (or length) of a particle.

The FBRM probe was introduced vertically into the flocculator, 10 mm above the PTFE shaker and 20 mm off-axis. The suspensions tested contained a flocculant dose of 34 g/t. The total measurement time before and during flocculation corresponded to 5 min.

The images of the aggregates over time were also recorded at a flocculant dose of 34 g/t in a PVM probe (particle vision measurement) model V819 Mettler-Toledo, which has an outer diameter of 19 mm and a 14 mm diameter sapphire window, which registers the images on a scale of 1075 × 825 µm under a resolution of 2 µm. This probe can acquire up to ten images per second.

### 2.3. Fractal Dimension and Density of the Aggregates

The structure of the aggregates can be described by their fractal dimension [23,24]. This is when the total volume of an aggregate, simplified as a sphere (L), increases with the cube of its diameter, while the mass (m) increases at a lower fractal power (see Equation (1)), where $D_{f}$ is the mass-length fractal dimension and takes a value between 1 and 3, considering 1 as a one-dimensional line and 3 as a solid sphere.

(1) $$m\propto L^{D_{f}}$$

The fractal dimension $D_{f}$ was calculated from the mathematical relationship used by Heath et al. [21], where the diameter of the aggregate and the hindered sedimentation are related. Chord length is used as an indicator of diameter, valid within the size range considered in the present study [25]. The relationship is expressed in Equation (2):

(2) $$U_{h}=\frac{d_{agg}^{2}g\left(\rho_{s}-\rho_{l}\right){\left(\frac{d_{agg}}{d_{p}}\right)}^{D_{f}-3}}{18µ}\;{\left(1-\phi_{s}{\left(\frac{d_{agg}}{d_{p}}\right)}^{3-D_{f}}\right)}^{4.65}$$

where $U_{h}$ is the hindered settling rate $\left[m/s\right]$; $d_{agg}$ and $d_{p}$ are the aggregate and particle diameters [m], respectively (approximated by the squared-weighted mean chord length), g is the acceleration of gravity $\left[9.81\;m/s^{2}\right]$, $\rho_{s}$ and $\rho_{l}$ correspond to the density of solid and liquid, respectively $\left[kg/m^{3}\right]$, μ is the viscosity of the fluid $\left[Ns/m^{2}\right]$, $\varphi_{s}$ is the volumetric fraction of solids $\left[v/v\right]$, and $D_{f}$ the fractal dimension.

The parameters g, $\rho_{s}$, $\rho_{l}$, μ, $\varphi_{s}$, $d_{p}$ are constant and known for all experiments. The diameter of the aggregate $d_{agg}$ and $D_{f}$ depend on the hydrodynamic conditions applied to the system. The hindered sedimentation rate is determined experimentally after 20 s of flocculation, as explained in Section 2.2. The $d_{agg}$ is provided by the mean squared weighted chord length obtained through the FBRM probe. Finally, the estimate $D_{f}$ is obtained from Equation (2) using least squares.

Obtaining $D_{f}$ allows estimation of the density of the aggregates from the Kranenburg derivation [26], Equation (3):

(3) $$\left(\rho_{agg}-\rho_{l}\right)=\left(\rho_{s}-\rho_{l}\right){\left(\frac{d_{agg}}{d_{p}}\right)}^{D_{f}-3}$$

where $\rho_{agg}$, $\rho_{s}$ and $\rho_{l}$ are the aggregate, solid, and liquid densities, respectively [kg/m^3^], $d_{agg}$ and $d_{p}$ are the aggregate and particle diameters, respectively $\left[m\right]$ that approximate the squared-weighted mean chord length obtained with the FBRM probe, and $D_{f}$ is the fractal dimension obtained from Equation (2).

## 3. Results and Discussions

### 3.1. Sedimentation of Flocculated Clay Tailings

This section analyzes the settling rate of four synthetic tailings, flocculated with anionic polyacrylamide of high molecular weight (SNF 704), changing the mineral composition and type of water. A mixture of kaolin/quartz and montmorillonite/quartz was considered the solid phase. The two types of water were industrial water (IW) and seawater (SW), prepared as indicated in Section 2.1.

**Figure 5:**
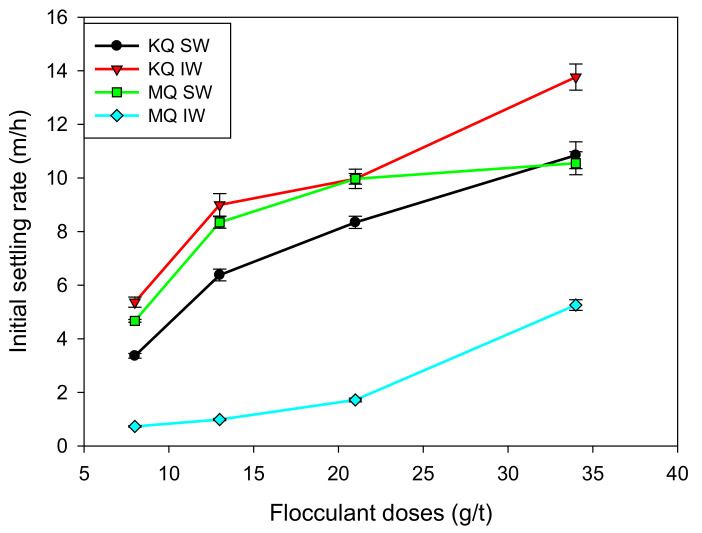
Initial settling rate at varied flocculant dosage for KQ and MQ synthetic tailings in SW and IW. Mixing rate 180 rpm, solids concentration 8 wt% (clay/quartz 10/90), flocculation time 20 s, pH 8.

Figure 5 shows that the settling rate increased with the flocculant dose within the flocculant dosage (8–34 g/t) range. The highest values were achieved with KQ tailings prepared in IW, with values between 5–14 m/h. When the tailings were prepared in a highly saline medium (SW), the coiling of the flocculant reduced the settling rate in all cases, obtaining values between 3–11 m/h. As reported by Quezada et al. [27], there were essential changes in the modes of interaction between the polymer and the mineral surface. HPAM interacts with the kaolin surface through the nitrogen of the acrylamide group with the deprotonated oxygen of the surface, while in quartz, the nitrogen of the acrylamide group interacts with the oxygen from the hydroxide on the surface. However, in a saline medium, cationic bridges predominate, favoring the interactions of the deprotonated oxygen of the acrylic group of HPAM with the deprotonated oxygen of the kaolin and with the hydroxide of the quartz surface. This fact directly impacts the quality of the supernatant water, as discussed in Section 3.5.

The tailings composed of montmorillonite and quartz (MQ) in IW reported low values of sedimentation velocity, situated in the range of 0.5–5.5 m/h for the doses used. The complexity of this clay, a product of its swelling nature, generates unfavorable sedimentation conditions, raising the pulp’s apparent volumetric concentration. In this context, the presence of seawater caused a significant increase, with sedimentation speeds in the range of 4–10 m/h. This effect responds to the compression of the ionic cloud between the clay layers [28]. The migration of cations towards the interior of the particles reduces the electrostatic repulsion between their layers. This reduces the interlaminar distance to the point of preventing the entry of water molecules into the clay, reducing the swelling effect.

### 3.2. Flocculation Kinetics of Flocculated Clay-Based Tailings

**Figure 6:**
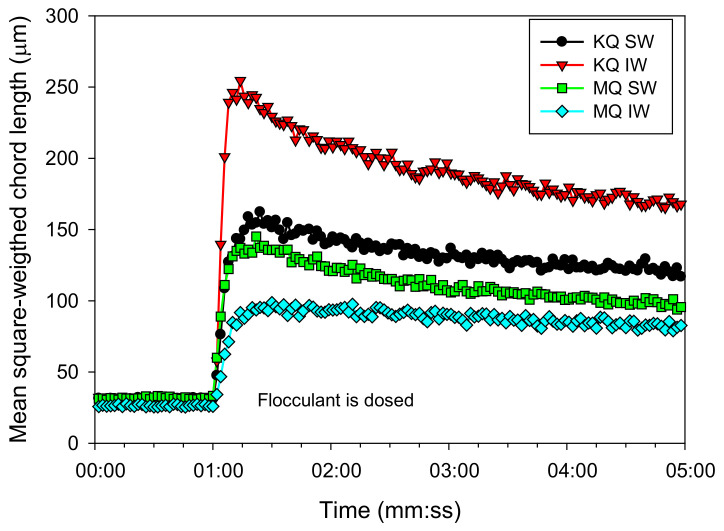
Flocculation kinetics of kaolin-quartz (KQ) and montmorillonite-quartz (MQ) tailings in SW and IW. Flocculant dose 34 g/t, mixing rate 180 rpm, solids concentration 8 wt% (clay/quartz 10/90), pH 8.

The use of FBRM allows for the analysis of the aggregation kinetics that results from the phenomena of aggregation, restructuring, and fragmentation of the structures. Figure 6 shows the results obtained for the four systems, that is, tailings with kaolin (KQ) and montmorillonite (MQ) contents, in industrial water (IW) and seawater (SW). The flocculation by the polymeric bridging, mediated by the flocculant SNF 704 (34 g/t), caused fast growth of aggregates, which reached a maximum size a few seconds after the flocculant was added. The largest floc size was obtained for KQ tailings in IW. The maximum size was 250 µm, achieved at 14 s of flocculation, while the same tailings in SW reached 150 µm at 16 s after the reagent was added. The result is consistent with the sedimentation tests, showing that seawater reduced the settling rates throughout the range of dosages.

In the case of MQ tailings, sizes smaller than those reported for KQ were obtained. However, the trend was reversed for the type of water, where a maximum size of 95 µm was reached with IW, without a subsequent (significant) decrease in size, but in SW, the maximum size achieved was 135 µm, obtained at 20 s. This phenomenon has been discussed in the literature, and the evidence indicates that the entry of divalent cations from seawater into the clay sheets reduces their volume in solution. Besides, the increase in cationic bridges between the surface and the flocculants favors flocculation [16,28].

### 3.3. Chord-Length Distributions (CLD)

**Figure 7:**
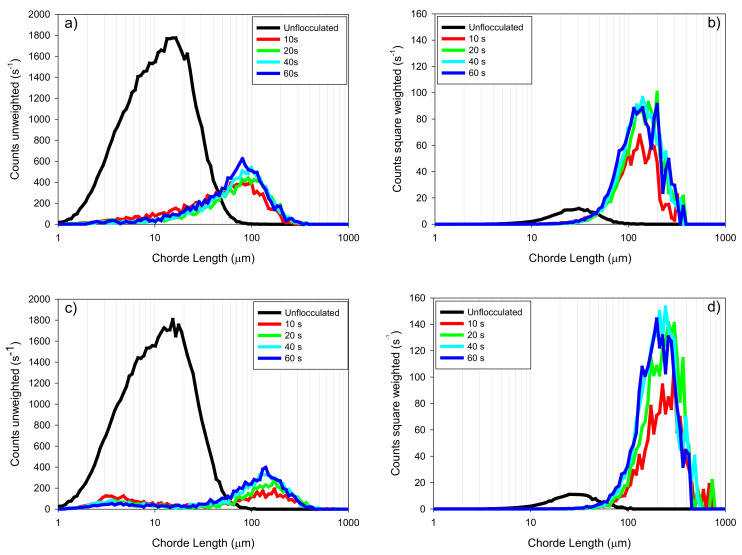
(**a**) unweighted chord length distribution (CLD) for KQ in SW, (**b**) square-weighted CLD for KQ in SW, (**c**) unweighted CLD for KQ in IW, (**d**) square-weighted CLD for KQ in IW. Flocculant dose 34 g/t, mixing rate 180 rpm, solids concentration 8 wt% (clay/quartz 10/90), pH 8. KQ = kaolin/quartz, SW = seawater, IW = industrial water.

Figure 7 shows the unweighted and quadratically weighted chord-length distributions for the tailings made up of kaolin (KQ) in both types of water. The unweighted distribution allows us to analyze the time evolution of the growth/fragmentation of flocs, especially in the smaller zone. In contrast, the square-weighted distributions better describe the changes in the coarse-aggregate region [29].

The fast aggregation is evidenced after only 10 s of reaction; there is a substantial change in the size distributions compared to the non-flocculated system. In the case of the unweighted distribution, specific differences are observed according to the type of water. When using SW (Figure 7a), the flocculant efficiently captures the primary particles (fines); however, in IW, the flocculant leaves particles without flocculating, and a clear bimodal distribution appears. One peak corresponds to the largest aggregates generated by a polymeric bridge, and the other peak represents the fine particles that were not captured (3–5 µm) [30]. As the flocculation elapses, the proportion of non-flocculated particles is reduced; that is, the fine particles needed more time to flocculate. For example, at 10 s of flocculation, the peak height is 130 s^−1^, but after 60 s, the peak height is 60 s^−1^. However, coarse aggregates were reduced in size, best observed in the quadratically weighted chord-length distribution (Figure 7b,d). It is possible that in the system prepared with SW, a binodal result did not appear because the high concentration of salts, especially divalent cations, improved the adsorption of HPAMs on the mineral surface, increasing the probability of adsorption of fine particles [31].

The square-weighted CLDs (Figure 7b,d) showed the sensitivity of coarse aggregates to fragmentation as a product of the hydrodynamic conditions during the mixing (180 rpm). Figure 7b, which corresponds to the system prepared in SW, shows the highest intensity of its peaks between 140 and 160 µm, in contrast to IW (Figure 7d) that presents them between 200 and 300 µm. The larger floc size responds to the greater stretching of the macromolecule in an environment with little salt content. The largest dimensions were obtained at the shortest flocculation time (10 s), while as the process advanced, the aggregate fragmentation reduced their size but increased the number. Figure 7b shows that, at 10 s, the peak does not exceed a count of 70 s^−1^, while at 20 s, the count is over 80 s^−1^. Figure 7d shows that the count at 10 s is around 80 s^−1^, but at 20 s, the value is close to 140 s^−1^.

**Figure 8:**
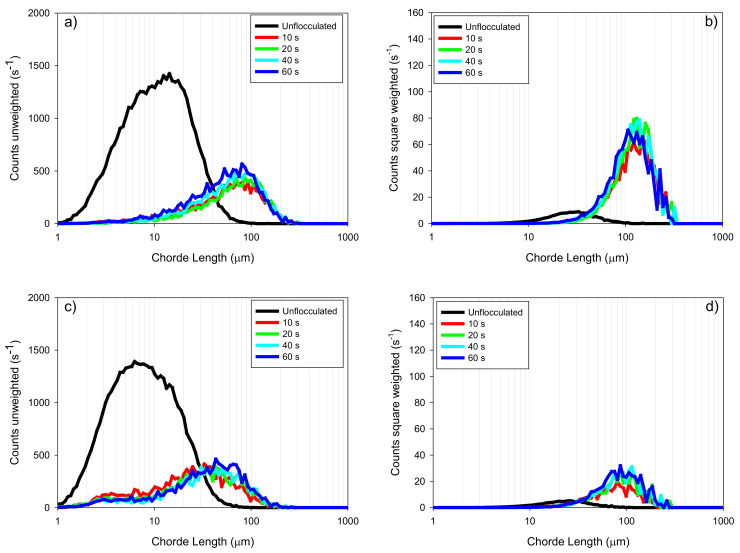
(**a**) unweighted CLD for MQ in SW, (**b**) square-weighted CLD for MQ in SW, (**c**) unweighted CLD for MQ in IW, (**d**) square-weighted CLD for MQ in IW. Flocculant dose 34 g/t, mixing rate 180 rpm, solids concentration 8 wt% (clay/quartz 10/90), pH 8. MQ = Na-montmorillonite/quartz, SW = seawater, IW = industrial water.

Figure 8 shows the unweighted (Figure 8a,c) and square-weighted (Figure 8b,d) CLD for the tailings made up of montmorillonite and quartz (MQ) in both types of water (IW and SW). The formation of larger aggregates was evidenced at 10 s of reaction, represented by the count peaks located around 80–120 µm for SW (Figure 8a) and 80–100 µm for IW (Figure 8c). The trend for time shows a behavior similar to what happened in the tailings made up of kaolin (Figure 7). There is a good capture of fine particles in seawater, while in industrial water, a bimodal distribution is suggested, with a region of non-flocculated fine particles from 3–7 µm. However, the square-weighted CLD does not show a significant displacement of the peak corresponding to the coarse zone; this is 150 µm for SW and 90 µm for IW, except for a subtle increase over time. The difference in the coarse aggregates was noticeable for both types of water, and consistent with the settling behavior described in previous sections, and can be explained according to DLVO theory for interpreting the phenomena occurring in the interlaminar layers of the montmorillonite.

### 3.4. Fractal Dimension

The aggregates formed by bridging flocculation are highly porous. It has been commonly accepted that aggregation modes follow fractal growth patterns that can be characterized through the fractal dimension $D_{f}$. This term is obtained through Equation (2), detailed in Section 2.3. The density of the aggregates is estimated from Equation (3).

**Figure 9:**
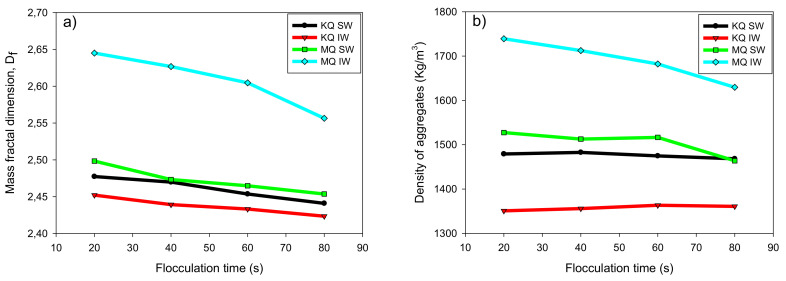
Fractal dimension (**a**) and density of aggregates (**b**) for KQ and MQ tailings in SW and IW. Flocculant dose 34 g/t, solids concentration 8 wt% (clay/quartz 10/90), mixing rate 180 rpm and pH 8.

Figure 9a,b shows the values of the fractal dimension $D_{f}$, and the density of the aggregates, $\rho_{agg}$, respectively, as a function of the flocculation time (20–80 s) and a flocculant dose of 34 [g/t]. It is observed that $D_{f}$ has an important dependence on the flocculation time for the four tailings studied, gradually decreasing in all cases. Aggregates formed by polymer bridging flocculation are expected to undergo irreversible breakage under prolonged mixing. An examination of Figure 9 would conclude that this coincides with the aggregates becoming increasingly irregular in shape and more porous in structure [18]. The lowest $D_{f}$ corresponded to KQ tailings, with the KQ-IW tailings presenting similar values of $D_{f}$ (2.45–2.42) to the KQ-SW tailings (2.47–2.44). This reduction in IW is related to larger aggregates formed in a medium with a low ionic charge. The tailings MQ in SW showed lower $D_{f}$ than IW, standing in (2.49–2.45). The reduction in $D_{f}$ in the presence of SW indicates that the aggregates are less compact and possibly with an open structure, far from a solid sphere.

It is important to note that the fractal dimension is a relevant parameter to define macroscopic properties that are of industrial interest, especially in thickening stages. Previous studies have shown a direct relationship of $D_{f}$ with sedimentation rates and rheological properties of flocculated suspensions. For example, Jeldres et al. [32] analyzed the $D_{f}$ of synthetic tailings composed of mixtures of quartz and kaolin in seawater. The authors observed a monotonic relationship between the fractal dimension of the aggregates and the yield stress of the sediment after a sedimentation process, with exponential trends, within the range analyzed. The growth patterns depended significantly on the agitation rates during flocculation, but tended to increase yield stress with the fractal dimension. This is also consistent with that presented by Deng and Davè [33], who established that a lower fractal dimension leads to a lower mechanical strength of the agglomerate.

The $\rho_{agg}$ (Figure 9b) was lower for KQ tailings throughout the range of flocculation time studied, with values between (1479–1468 kg/m^3^) for KQ-SW and (1360–1350 kg/m^3^) for KQ-IW. Previous studies have shown that aggregates larger than 100 µm, sometimes called macro-aggregates, generally contain higher proportions of intra-aggregate fluid than smaller aggregates [34,35], which greatly reduces the density of the aggregates until it is only slightly greater than continuous fluid [36]. This is also because the polyelectrolyte is extended in the medium, with few points of contact on the mineral surfaces, considering the anionic nature of the particles and functional groups of the polymer. It is expected that the configuration acquired by the adsorbed flocculant is through loops; however, the reduction in the ionic cloud and additional cation bonds that occurs in seawater changes the flocculant adsorption mode, favoring train configurations [11], which lead to more compact aggregates. This justifies the low density of the aggregates and $D_{f}$ for the KQ-IW tailing, which obtained the largest particle aggregation size of approximately 250 [µm], as evidenced in Figure 6.

### 3.5. Images of Non-Flocculated and Flocculated Particles

**Figure 10:**
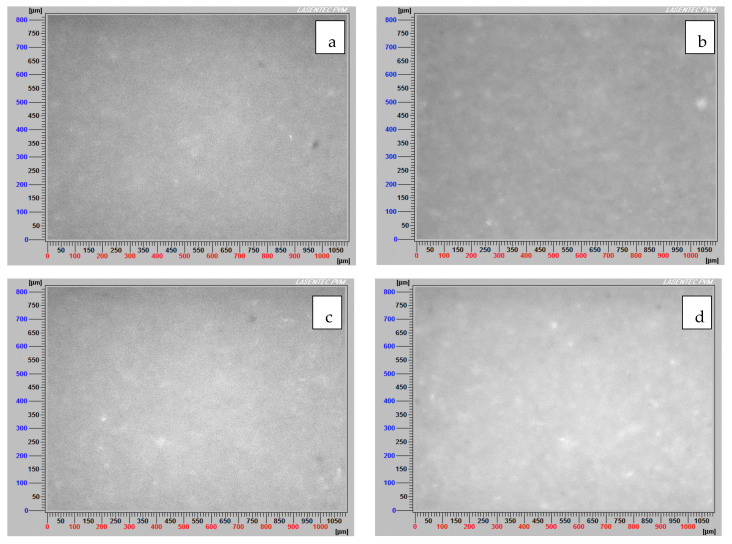
Images of non-flocculated tailings (**a**) KQ-SW, (**b**) KQ-IW, (**c**) MQ-SW, (**d**) MQ-IW. Mixing rate 180 rpm, solids concentration 8 wt% (10/90 clay/quartz), pH 8.

Figure 10a–d display the four non-flocculated tailings: KQ-SW, KQ-IW, MQ-SW, and MQ-IW, respectively. These are at a mixing rate of 180 rpm and a solid of 8 wt%. There are no significant changes in the images obtained with the PVM probe. Most of the particles are above or below the instrument’s resolution, including partially coagulated particles, such as the particles present in MQ-SW tailings; consequently, the finer particles can shield the appearance of the bigger ones.

**Figure 11:**
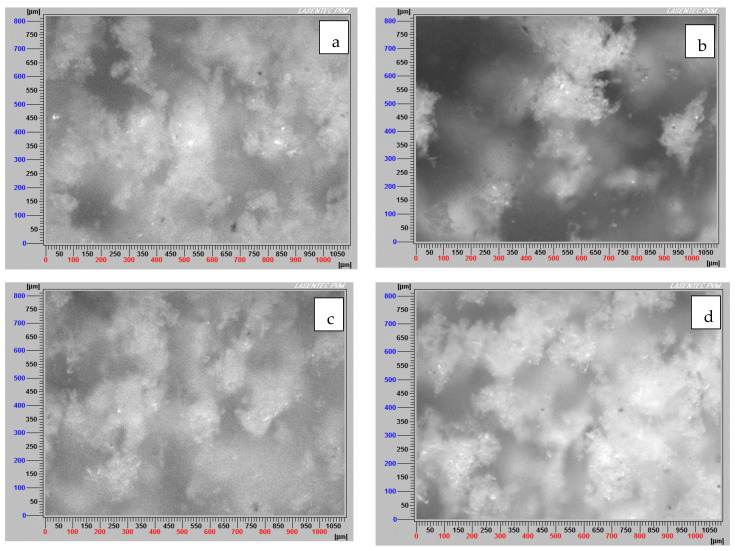
Images of flocculated tailings (**a**) KQ-SW, (**b**) KQ-IW, (**c**) MQ-SW, (**d**) MQ-IW. Flocculant dose 34 g/t, mixing rate 180 rpm, solids concentration 8 wt% (clay/quartz 10/90), flocculation time 20 s approx., pH 8.

On the other hand, Figure 11a–d show images of the four tailings, but flocculated. In Figure 11b, aggregates can be seen that vary in their characteristics and shape. While some are oversized without having a tall aspect ratio, others are quite elongated. Figure 11a,c,d show smaller aggregates with a slightly more elongated appearance. Figure 11d shows smaller aggregates, due to montmorillonite’s swelling property at low salt concentrations, and also to their difficulty in flocculating.

## 4. Conclusions

The present research examined the phenomena of the flocculation and sedimentation of clay-based tailings, considering two types of clays: kaolin and montmorillonite. The assays were performed in two kinds of waters, prepared synthetically: seawater and industrial water.

The effect of the type of water on flocculation differs between tailings. In the first case, for quartz/kaolin, the highest performance was obtained in industrial water, with more significant aggregates and settling rates. The high salinity of the seawater rolled up the flocculant, reducing its ability to bridge particles, but at the same time, led to more compact aggregates and higher density. This was promoted by the differences in the adsorption modes of the flocculant on the mineral surfaces. Loop configurations are preferred in industrial water, while train configurations are favored in a highly saline medium like seawater.

On the other hand, the tailings composed of quartz and montmorillonite reversed this trend with respect to the type of water. The best flocculation was produced in seawater, although the flocculant was coiled. The swelling nature of this clay in industrial water harms flocculation, and high flocculant doses are required to promote particle aggregation. The high cation concentrations reduced the swelling phenomena, with a substantial improvement in the aggregation by the polymeric bridging.

The type of water impacted the efficiency of primary particles aggregation. The trials in industrial water generated a portion of non-flocculated particles, independent of the type of tailings observed through a bimodal distribution in the unweighted chord-length distribution. At the same time, by making the studies in seawater, there were no perceptible fractions of non-flocculated particles. The formation of additional bonds due to the increased cation concentration favored the agglomeration of finer primary particles.

## Figures and Tables

**Table 1:** Chemical composition of synthetic seawater.

Salt	Concentration (g/L)
NaCl	24.53
MgCl_2_·6H_2_O	11.10
Na_2_SO_4_	4.09
CaCl_2_	1.16
KCl	0.69
NaHCO_3_	0.20
KBr	0.10
H_3_BO_3_	0.03

**Table 2:** Physical and chemical properties of seawater and industrial water at 23 °C and 1 atm.

	Seawater	Industrial Water
pH	7.97	7.40
Density (kg/m^3^)	1024	998.5
Electric Conductivity (mS/cm)	53.1	2.24
Salinity (PSU)	33.81	1.10

## Data Availability

The data presented in this study are available on request from the corresponding author.
